# Highly Efficient Photoelectrocatalytic Reduction of CO_2_ to Methanol by a p–n Heterojunction CeO_2_/CuO/Cu Catalyst

**DOI:** 10.1007/s40820-019-0354-1

**Published:** 2020-01-09

**Authors:** Zhengbin Pan, Ershuan Han, Jingui Zheng, Jing Lu, Xiaolin Wang, Yanbin Yin, Geoffrey I. N. Waterhouse, Xiuguo Wang, Peiqiang Li

**Affiliations:** 1 Tobacco Research Institute of Chinese Academy of Agricultural Sciences (CAAS), Qingdao, 266101 People’s Republic of China; 2 College of Chemistry and Material Science, Shandong Agricultural University, Tai’an, 271018, People’s Republic of China; 3 School of Chemical Sciences, The University of Auckland, Auckland, 1142 New Zealand

**Keywords:** CO_2_ reduction, Photoelectrocatalysis, p–n heterojunction, Cerium oxide, Copper oxide

## Abstract

Photoelectrocatalytic reduction of CO_2_ to fuels has great potential for reducing anthropogenic CO_2_ emissions and also lessening our dependence on fossil fuel energy. Herein, we report the successful development of a novel photoelectrocatalytic catalyst for the selective reduction of CO_2_ to methanol, comprising a copper catalyst modified with flower-like cerium oxide nanoparticles (CeO_2_ NPs) (a n-type semiconductor) and copper oxide nanoparticles (CuO NPs) (a p-type semiconductor). At an applied potential of − 1.0 V (vs SCE) under visible light irradiation, the CeO_2_ NPs/CuO NPs/Cu catalyst yielded methanol at a rate of 3.44 μmol cm^−2^ h^−1^, which was approximately five times higher than that of a CuO NPs/Cu catalyst (0.67 μmol cm^−2^ h^−1^). The carrier concentration increased by ~ 10^8^ times when the flower-like CeO_2_ NPs were deposited on the CuO NPs/Cu catalyst, due to synergistic transfer of photoexcited electrons from the conduction band of CuO to that of CeO_2_, which enhanced both photocatalytic and photoelectrocatalytic CO_2_ reduction on the CeO_2_ NPs. The facile migration of photoexcited electrons and holes across the p–n heterojunction that formed between the CeO_2_ and CuO components was thus critical to excellent light-induced CO_2_ reduction properties of the CeO_2_ NPs/CuO NPs/Cu catalyst. Results encourage the wider application of composite semiconductor electrodes in carbon dioxide reduction.

**Electronic supplementary material:**

The online version of this article (10.1007/s40820-019-0354-1) contains supplementary material, which is available to authorized users.

## Highlights

- Flower-like CeO_**2**_ nanoparticles enhance the performance of CuO nanoparticle/Cu.
- The system benefits from the heterojunction between p-type CuO and n-type CeO_2_.
- The selective reduction of CO_2_ to methanol on the target catalyst is studied.

**Electronic supplementary material:**

The online version of this article (10.1007/s40820-019-0354-1) contains supplementary material, which is available to authorized users.

## Graphical abstract

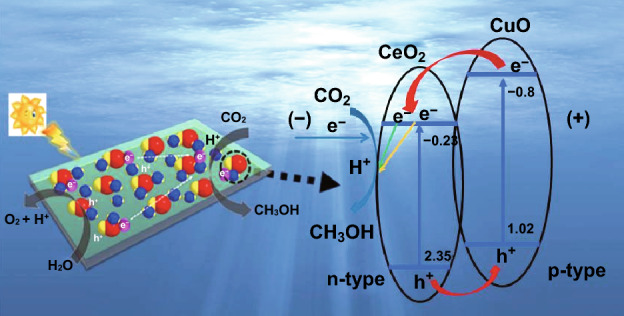

## Introduction

Since the beginning of the industrial revolution in the late 1700s, humans have heavily relied on cheap and abundant fossil fuels as an energy source. Indeed, modern societies are still very heavily reliant on fossil fuels for energy and as a feedstock for the manufacture of commodity chemicals. However, fossil fuels are a non-renewable energy source, meaning that they will likely be exhausted over the next few hundred years, motivating the development alternative renewable energy technologies (especially solar and wind) as a means of achieving energy supply security. A further downside of using fossil fuels for energy is the associated CO_2_ emissions, which directly promote global warming via the greenhouse effect [1–4]. However, if viewed from another perspective, the CO_2_ produced via combustion of fossil fuels or biomass sources represents a cheap C_1_ building block (i.e., a resource to be exploited rather than an annoying waste product) [5, 6]. Accordingly, over the past decade, and enormous amount of research effort has been directed toward the development of efficient technologies for converting CO_2_ into valuable chemical and fuels via processes such as catalytic hydrogenation [7, 8], photocatalytic (PC) reduction [9, 10], electrocatalytic (EC) reduction [11, 12], photoelectrocatalytic (PEC) reduction [13, 14], and biotransformation to solve environmental and energy issues [15, 16]. Among these, EC, PC, and PEC are perhaps the three most promising approaches for future large scale CO_2_ reduction. In EC, CO_2_ is reduced via the transfer of electrons and also protons to CO_2_, though most metal electrodes suffer from high overpotentials for CO_2_ reduction, and thus produce large amounts of H_2_ along with any products of CO_2_ reduction (i.e., the electrodes generally have poor selectivity for CO_2_ reduction) [17, 18]. In CO_2_ reduction by semiconductor photocatalysis, light irradiation causes electrons to be promoted from the semiconductor valence band into the conduction band (CB), thus giving rise to charge carriers capable of reducing CO_2_ to CO and other products when the semiconductors valence band (VB) and CB are suitably positioned relative to the redox processes needed for CO_2_ reduction and typically water oxidation, respectively [19]. However, the efficiency and performance of most photocatalysts for CO_2_ reduction are limited by bulk recombination of photoexcited electrons and holes. In this context, PEC is a particularly promising method for CO_2_ reduction, wherein an applied potential is used to prevent the bulk recombination of bulk photoelectrons and holes, thereby increasing the availability of photoexcited electrons for reaction with CO_2_. Various semiconductors have been studied in relation to PC and PEC CO_2_ reduction. In order to maximize solar spectrum utilization, it is desirable to use a semiconductor with a narrow band gap that absorbs in the visible regime [20]. However, the narrower the band gap, the more rapid the electron–hole pair recombination, and hence many narrow band gap semiconductors show poor PC efficiencies for CO_2_ reduction. Thus, PEC systems are generally preferred, with various approaches being adopted to enhance the conversion efficiency of PEC catalysts for CO_2_ reduction including the introduction of co-catalysts [21, 22], bulk doping of the semiconductor with metal cations to increase the light absorption range and/or to introduce electron traps [23, 24], p–n junction construction [25], among others. Furthermore, by nanostructuring semiconductor PC or by creating hierarchical semiconductor arrays, materials with large specific surface areas and abundant active sites for adsorbing CO_2_ can be created, thereby facilitating CO_2_ reduction under direct sunlight or simulated sunlight [26].

Copper oxide-based semiconductors have received much attention in relation to the catalytic reduction CO_2_. CuO has excellent EC activity for CO_2_ reduction, exhibiting very high selectivity to CH_3_OH [27, 28]. Further, Gusain et al. [29] demonstrated that reduced graphene oxide–copper oxide nanocomposites were able to convert CO_2_ to CH_3_OH photocatalytically under visible light irradiation. Kim et al. [30] developed a highly efficient p–n–p Cu_2_O/S-TiO_2_/CuO heterojunction for the photocatalytic conversion of CO_2_ to CH_4_. Although CuO can readily be photoexcited using visible light, rapid recombination of photogenerated electrons and holes limits its photocatalytic activity [31]. Combining CuO with other semiconductors through heterojunction is effective in suppressing electron–hole pair recombination, offering a pathway to greatly improve the PC performance of CuO. Other strategies for suppressing electron–hole pair recombination include the addition of suitable co-catalysts or photosensitizers that can supply electrons or remove holes effectively from CuO, or alternatively enhance the absorption incoming visible light [32]. CeO_2_ is an inexpensive rare earth semiconductor that finds widespread use in solar cells, optical devices, sensors, and sunscreen materials [33, 34]. It is also widely used in catalysis as a support due to its outstanding oxygen storage behavior, which arises from valence change switching between Ce^3+^ and Ce^4+^ [35, 36]. Considering the relative positions of the conduction and valance bands of n-type CeO_2_ and p-type CuO, we hypothesized that the construction of a CeO_2_/CuO heterojunction should be highly beneficial for improving the PC and PEC performance for CO_2_ reduction. Under light irradiation, electrons photoexcited into the CB of CuO would be transferred into the CB of CeO_2_, whereas holes (h^+^) created in the VB of CeO_2_ under light irradiation would migrate into the VB of CuO. Via such exchange of electrons holes between CuO and CeO_2_, more charge carriers should be available to drive CO_2_ reduction to methanol (the dominant product for CO_2_ reduction over CuO-based catalysts). To date, very little work has been done relating to the application of CeO_2_/CuO heterojunctions in solar-driven CO_2_ reduction to fuels, motivating a detailed investigation.

Herein, we systematically explored the performance of a CeO_2_/CuO/Cu catalyst system for PEC CO_2_ reduction under visible light. Flower-like n-type CeO_2_ nanoparticles were grown on a p-type CuO NPs/Cu foil catalyst by electrodeposition, with the resulting heterojunction catalyst (denoted herein as CeO_2_ NPs/CuO NPs) then being subjected to detailed physicochemical and electrochemical characterization, along with PEC CO_2_ reduction tests in a CO_2_-saturated aqueous KHCO_3_ solution. The overarching aim of the research was to establish whether CeO_2_ addition could significantly enhance the PEC performance of CuO-based catalyst for reduction CO_2_ to CH_3_OH.

## Experimental Section

### Preparation of the CuO NPs Catalyst

All reagents were supplied by Aladdin Reagent Co., Ltd. A pure copper foil (99.99% Cu, 50 × 20 × 1 mm^3^) was polished with coarse sandpaper, then metallographic sandpaper, to achieve a mirror finish. The polished Cu foil was washed repeatedly with anhydrous ethanol and then air-dried. The Cu foil was then anodized in an ethylene glycol–water solution (volume ratio 97:3) containing 0.25wt% NH_4_F. A polished Ti foil (99.99% Ti, 50 × 20 × 1 mm^3^) was used as the cathode. The separation between the anode and cathode was 1 cm, and the temperature of electrolyte maintained at 20 °C. Under magnetic stirring, CuO NPs were formed by holding the Cu foil electrode at a potential of 30 V for 1.5 h. The prepared electrode was then washed with distilled water before being heated from room temperature to 300 °C in a tube furnace under O_2_. The catalyst was held at 300 °C for 3 h and then cooled to 20 °C at a rate of 1 °C min^−1^ [13]. The obtained catalyst is denoted herein as CuO NPs.

### Preparation of the Flower-like CeO_2_ NPs/CuO NPs Composite Catalyst

Flower-like CeO_2_ NPs were subsequently deposited on the CuO NPs catalyst via an electrochemical method that used a three-electrode system, comprising the CuO NPs catalyst as the working electrode, a Pt wire as the counter electrode and a saturated calomel electrode (SCE) as the reference electrode. The flower-like CeO_2_ NPs were introduced by electrodeposition at a constant current density of 2 mA cm^−2^ for 2 h. An aqueous electrolyte containing 10 mM Ce(NO_3_)_3_·6H_2_O, 50 mM KCl, and 50 mM NH_4_Cl, with the electrolyte temperature maintained at 70 °C [37]. The obtained catalyst, denoted herein as the flower-like CeO_2_ NPs/CuO NPs, was washed with distilled water and air-dried at room temperature.

### Catalysts Characterization

The surface morphologies of the catalysts were examined using scanning electron microscopy (SEM, Hitachi SU8010). The particle size was calculated by the Nano Measurer 1.2 software. The microscope was operated at an acceleration voltage of 5.0 kV for the imaging. High-resolution transmission electron microscopy (HRTEM) analyses were performed on a JEM-2100 microscope operating at 200 kV. The crystal structure of the samples was probed by X-ray diffraction (XRD) on a Rigaku D/MAX-rA diffractometer (Japan) equipped with a Cu-Kα source (λ = 0.154178 nm,). X-ray photoelectron spectroscopy (XPS) measurements were performed on a ESCALAB 250 equipped with a monochromatic Al Kα source (hν = 1486.7 eV). UV–visible diffuse reflectance spectra (UV–Vis DRS) were collected on a UV–Vis spectrophotometer (TU-1901. Beijing Purkinje General Instrument Co., Ltd.), and the wavelength ranged from 400 to 700 nm. Dielectric constants were measured at room temperature using a TH2826 LCR digital bridge (Shanghai Double Asa Electronics Co., Ltd.) operating at 2 MHz. The electrochemical properties of the catalysts were evaluated using a CHI660D potentiostat (Shanghai Chenhua Instrument Co., Ltd.).

### Photoelectrocatalytic CO_2_ Reduction Experiments

**Scheme 1:**
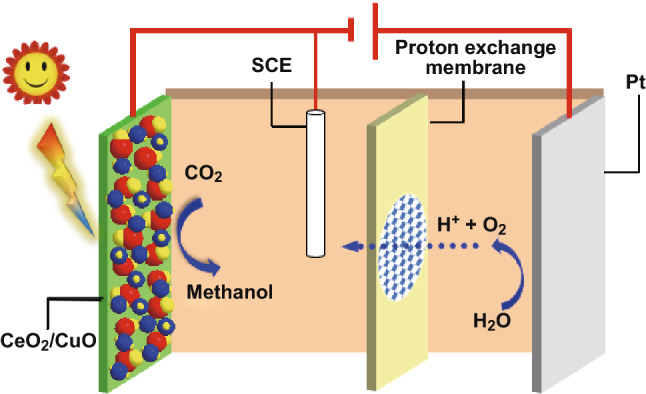
The PEC cell for CO_2_ reduction

PEC CO_2_ reduction experiments were conducted in a quartz reaction cell containing a three-electrode array comprising the CeO_2_ NPs/CuO NPs composite catalyst as the working electrode, a Pt plate as the counter electrode and a saturated calomel electrode (SCE) as the reference electrode. The cell was interfaced with a CHI660D potentiostat and a circulating water device to maintain a constant temperature of 25 °C. Scheme 1 shows a schematic of the PEC cell used for reduction CO_2_ reduction. The cathode electrolyte was aqueous 0.1 mol L^−1^ KHCO_3_ (40 mL), with CO_2_ gas bubbled through the cathode electrolyte at a rate of 40 mL min^−1^ during the reaction. In fresh form of 0.1 mol L^−1^ KHCO_3_, it has pH (8.34), but the reaction is carried out under CO_2_, so pH change to 6.8. The LSV is affected by different pH, experiments have been done under pH = 7.0 and pH = 9.0, and the impact was also calculated, which is 0.12%. So the proton reduction has almost no impact on the experimental conclusion [38]. A 500-W xenon lamp (420 ≤ λ ≤ 800 nm, 100 mW cm^−2^) was used for the PEC CO_2_ reduction tests, which were performed over a testing period of 6 h. The qualitative and quantitative analysis of products was performed by gas chromatography. The gas chromatograph (GC-9A, Shimadzu) was equipped with an air pump and hydrogen generator, FID and TCD detectors, and a Porapak Q (80–100) glass packed column. The column temperature was maintained at 180 °C and the detector temperature at 200 °C. The carrier gas used was high purity N_2_ (flow rate 30 mL min^−1^).

## Results and Discussion

### Characteristics of the CuO NPs and CeO_2_ NPs/CuO NPs Catalysts

**Fig. 1:**
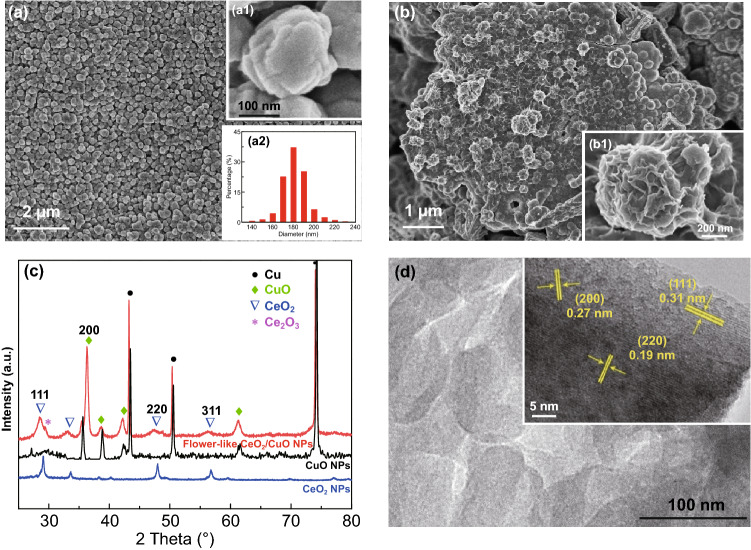
SEM images of CuO NPs at **a** low magnification and **a**_**1**_ high magnification. **a**_**2**_ Particle size distribution for CuO NPs. SEM images for flower-like CeO_2_ NPs at **b** low magnification and **b**_**1**_ high magnification. **c** XRD patterns for CuO NPs and flower-like CeO_2_ NPs/CuO NPs. **d** HRTEM image of CeO_2_ NPs. The inset shows a high magnification image, revealing lattice fringes characteristic for CeO_2_

The surface morphologies and structural properties of the CuO NPs and CeO_2_ NPs/CuO NPs catalysts were examined by SEM, TEM, and XRD, with data for these electrodes being summarized in Fig. 1. As shown in Fig. 1a, anodizing the surface of the Cu foil was effective in covering the surface of the foil with CuO NPs. The CuO NPs were pseudo-spherical and relatively uniform in size, with the average size around 180 nm (Fig. 1a_2_). XRD results confirmed the formation of CuO NPs (Fig. 1c). Flower-like CeO_2_ NPs were grown on the CuO NPs catalyst by electrodeposition (Fig. 1b). The flower-like CeO_2_ NPs morphology with thin petals was expected to be near ideal for promoting PEC CO_2_ reduction on the hybrid heterojunction catalyst, offering a very large specific surface area for CO_2_ adsorption and reduction (Fig. S1). XRD results further confirmed the successful growth of nanocrystalline CeO_2_ on the CuO NPs catalyst (Fig. 1c). Diffraction peaks appearing at 2θ = 28.58, 33.13, 47.56, and 56.43 could readily be assigned to the (111), (200), (220), and (311) reflections of CeO_2_ (JCPDS No. 65-5923). In addition, a small amount of Ce_2_O_3_ was detected, implying that the modified electrode contained an abundance of Ce^3+^/Ce^4+^ species and thus likely strong oxygen storage capacity [39]. The structure of the flower-like CeO_2_ NPs was examined further using HRTEM. Figure 1d shows that the sample was composed of CeO_2_ nanocrystals. The inset in Fig. 1d shows lattice fringes with spacings of 0.31, 0.19, and 0.27 nm, corresponding to the interlayer distance between the (111), (220), and (200) lattice planes, respectively, of cubic CeO_2_ [40]. These results are consistent with the XRD findings (Fig. S2). Previous studies have shown the CeO_2_ (111) plane to be very important for PC applications, as it is thermodynamically stable and very sensitive to light [35, 36].

**Fig. 2:**
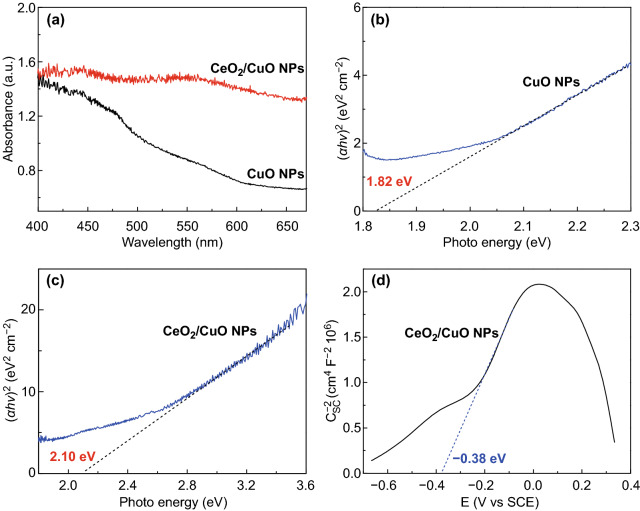
**a** UV–Vis absorbance spectra for CuO NPs and flower-like CeO_2_ NPs/CuO NPs. Tauc plots for **b** CuO NPs and **c** flower-like CeO_2_ NPs/CuO NPs. **d** Mott–Schottky plots for flower-like CeO_2_ NPs/CuO NPs

The visible light absorption characteristics of the as-prepared CuO NPs and flower-like CeO_2_ NPs/CuO NPs catalysts were examined by UV–Vis DRS (Fig. 2a). The flower-like CeO_2_ NPs/CuO NPs catalyst exhibited absorption across the visible region than CuO NPs catalyst, as expected since nanocrystalline CeO_2_ is optically active at visible wavelengths (a pale yellow color). Band gaps (*E*_g_) for the different electrodes were estimated using the Tauc equation (Eq. 1) [41]:

1 $$\alpha hv = A(hv - E_{\text{g}} )^{n}$$

here *α* is the reciprocal of the absorption length, *hv* is the photon energy, and *A* is a constant. For direct band gap transitions, the index *n* = 0.5 and for indirect band gap transition *n* = 2. The band gap of the CuO NPs catalyst was calculated by applying a linear fit to a plot of *(αhv*)^0.5^ and *hv* experimental curves, with the analysis yielding an *E*_g_ of 1.82 eV (Fig. 2b). This value is in good accord with prior literature reports for CuO [32]. For the flower-like CeO_2_ NPs/CuO NPs catalyst, the estimated *E*_g_ value was 2.10 eV (Fig. 2c), representing the weighted sum of the band gap values of CuO and CeO_2_. The data in Fig. 2 confirm that both electrodes showed a good optical response at visible wavelengths, as required for visible light-driven PC and PEC processes.

**Scheme 2:**
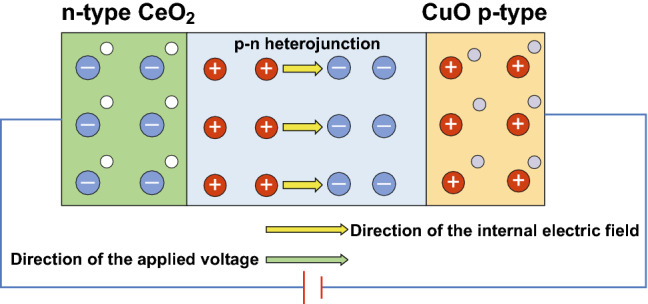
Charge separation under an applied the voltage in the p–n junction flower-like CeO_2_ NPs/CuO NPs system

When a semiconductor is in contact with an electrolyte, a space charge layer will be formed in the semiconductor surface due to the difference in Fermi level between the bulk and the surface (resulting in band bending), while a Helmholtz layer will be formed on the electrolyte side of the semiconductor/electrolyte interface. For such a system where a semiconductor is in contact with an electrolyte, a capacitance (*C*) results through coupling of the space charge layer capacitance (*C*_sc_) in series with the solution Helmholtz layer capacitance (*C*_H_) (note: C_H_ is usually negligible relative to *C*_sc_). Changing the polarization potential (*E*) of a semiconductor can change the space charge layer capacitance of the semiconductor, which can be described by the Mott–Schottky equation (Eq. 2) [42]:

2 $$\frac{ 1}{{C_{SC}^{ 2} }} = \frac{ 2}{{\varepsilon_{0} \varepsilon_{\text{r}} N_{A} A^{ 2} }}\left( {E - E_{\text{fb}} - \frac{{{\text{k}}T}}{\text{e}}} \right)$$

here *C*_sc_, *N*_A_, *ε*_0_, A, ε_r_, *E*, *k*, *T*, *E*_fb_ and *e* are the space charge layer capacitance, doping concentration, vacuum dielectric constant, the contact area between the electrode and the solution, dielectric constant of the semiconductor, polarization potential, the Boltzmann constant, absolute temperature, the flat band potential, and the electric charge, respectively. By plotting *C*_sc_ versus the polarization voltage *E*, a straight-line section is obtained. Extending the tangent to that straight-line section to the polarization voltage axis yields obtain the flat band potential *E*_fb_ of a material. Figures 2d and S3 show Mott–Schottky plots for the flower-like CeO_2_ NPs/CuO NPs catalyst and CuO NPs catalyst, respectively. The slope of the first half is positive, while the slope of the second half is negative, it shows p–n semiconductor characteristics after the CeO_2_ NPs deposited on the CuO NPs (Fig. 2d). *E*_fb_ of CuO NPs and CeO_2_ NPs/CuO NPs estimated from the plots were 0.82 eV (Fig. S3) and − 0.38 eV (Fig. 2d), respectively. The valence band maximum (VBM) of p-type semiconductor is approximately located at 0.2 eV below the flat band potential, so the VB of the CuO NPs is located at 1.02 eV [43]. Taking the fermi level for each electrode and using the Mott–Schottky equation, the acceptor concentrations (*N*_A_) for the CuO NPs catalyst and flower-like CeO_2_ NPs/CuO NPs catalyst were calculated to be 3.47 × 10^−5^ and 2.93 × 10^4^ m^−3^, respectively. The carrier concentration for the flower-like CeO_2_ NPs/CuO NPs catalysts was thus a remarkable 10^8^ times higher than that of the CuO NPs electrode. These data provide convincing evidence that the decoration of the CuO NPs catalyst with flower-like CeO_2_ NPs was effective in enhancing the separation of photogenerated electrons and holes. To explain this, the intrinsic properties of CuO (p-type conductor) and CeO_2_ (n-type conductor) need to be considered. On electrodepositing CeO_2_ onto the CuO NPs catalyst, p–n heterojunctions are formed at the interface between the two components (Fig. 2d). The experimental data suggest that when an external voltage is applied, the internal charge within the heterojunction actually opposed the applied external voltage, thus increasing the conductivity in the interfacial region [44–46]. Thus, creation of p–n heterojunctions is highly advantageous for promoting interfacial charge transfer and enhancing charge carrier mobilities [47]. Under the conditions used in the PEC CO_2_ reduction tests below, a negative voltage (− 1.0 V) was used, in which case the net flow of electrons will be from CuO to CeO_2_ (as depicted in Scheme 2), with CeO_2_ acting as the adsorption and reduction site for CO_2_.

**Fig. 3:**
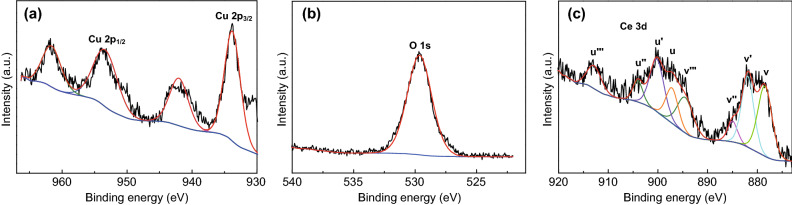
XPS characterization of the flower-like CeO_2_ NPs/CuO NPs composite catalyst. **a** Cu 2p region, **b** O 1s region, and **c** Ce 3d region

In order to further probe the excellent properties of the flower-like CeO_2_ NPs/CuO NPs system, XPS was applied to examine the chemical speciation of Cu, O, and Ce in the electrode. Figure 3a shows the Cu 2p XPS spectrum of the catalyst. The spectrum shows a set of peaks in a 2:1 area ratio (readily assigned to Cu p_3/2_ and Cu 2p_1/2_ signals, respectively), along with a corresponding shake-up satellites at higher binding energies, indicating the presence of Cu^2+^ (3 d^9^). The binding energy of the Cu 2p_3/2_ peak at 933.8 eV is also consistent with Cu^2+^ in CuO. The O 1 s peak at 529.8 eV (Fig. 3b) contains unresolved contributions from CuO (529.6 eV) and CeO_2_ (529.2 eV). The Ce 3d spectrum (Fig. 3c) showed a multitude of peaks, which could be assigned to Ce^4+^ and Ce^3+^ species following the description by Burroughs et al. [48] wherein *v* and *u* represent different 3d_5/2_ and 3d_3/2_ states, respectively. The characteristic peaks of Ce^4+^ are labeled *v, v”, v”‘, u, u”* and *u*”‘. The peaks labeled *v*’ and *u*’ are associated with Ce^3+^ (3d_5/2_ and 3d_3/2_, respectively), indicating the presence of oxygen vacancies on the surface of the CeO_2_ [49]. The percentage of Ce^3+^ in the flower-like CeO_2_ NPs/CuO NPs system was estimated to be around 34.82%, with the balance being Ce^4+^. The valence of Ce is analyzed in Table 1, and the result is consistent with the CeO_2_ existing in the form of CeO_2-x_.

Based on the aforementioned experimental studies of the flower-like CeO_2_ NPs/CuO NPs catalyst, mechanisms can be proposed for charge separation and thus CO_2_ reduction under EC, PC, and PEC regimes. On forming a CeO_2_/CuO heterojunction, an internal electric field will form at the interface between n-type CeO_2_ and p-type CuO, which inhibit the transfer of carriers between the two semiconductors.

If an external voltage is applied (under EC conditions as shown in Scheme 2) in the same direction as the internal electric field, the internal electric field should have become larger, and the conductivity poorer. However, we found that the internal electrical field of the CeO_2_ NP/CuO NPs was actually opposed to the external voltage (Scheme 2), increasing the interfacial conductivity, resulting in electron transfer to the CeO_2_ component. Accordingly, in the presence of CO_2_, rapid reduction to methanol on CeO_2_ will occur aided by protons coming from the anode (see CO_2_ tests below).

**Scheme 3:**
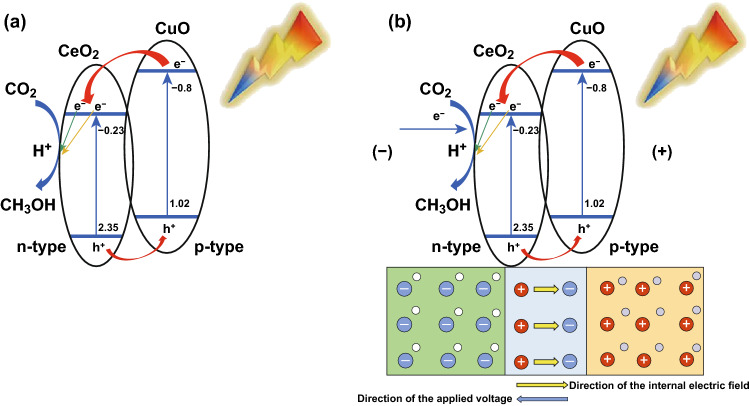
Proposed mechanisms for **a** PC CO_2_ reduction, and **b** PEC CO_2_ reduction on the flower-like CeO_2_ NPs/CuO NPs composite catalyst. The mechanism of EC CO_2_ reduction is shown in Scheme 2

Under light irradiation (no voltage applied, i.e., the PC regime, Scheme 3a), electrons will be photoexcited from the VB to the CB of both CuO and CeO_2_. Electrons photoexcited in CuO will be transferred to CeO_2_ since the CB of CeO_2_ (− 0.23 eV) is more positive than that of the CuO (− 0.80 eV) [50]. Conversely, holes created in the VB of CeO_2_ will migrate into the VB of CuO. It results in the suppression of electron–hole pair recombination and an increase in the availability of electrons for reducing CO_2_ on the CeO_2_ nanoparticles.

When light and electricity are applied simultaneously (Scheme 3b), the concentration of electrons reaching CeO_2_ will be further enhanced through the coupling of processes described in Scheme 2 and 3a. Accordingly, PEC CO_2_ reduction, which synergistically combines the advantages of EC and PC systems, was expected to offer the best performance for CO_2_ reduction to CH_3_OH, which was confirmed by the experiment below.

**Fig. 4:**
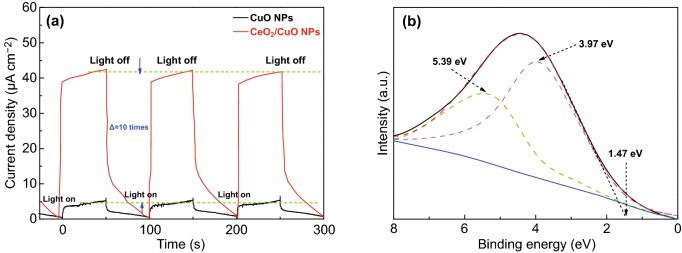
**a** Instantaneous photocurrent density of catalyst, **b** VB photoemission spectrum (solid line) and Gaussian fit (dashed line) of catalyst

The instantaneous photocurrent density excited by visible light provides a useful indication of the efficiency of the separation of photogenerated electrons and holes on the surface of a semiconductor photocatalyst. The higher the instantaneous photocurrent density, the more sensitive the photocatalyst is to visible light and the greater the separation of photogenerated electrons and holes. Figure 4a shows instantaneous photocurrent density changes for the CuO NPs and CeO_2_ NPs/CuO NPs catalysts over several light-on and light-off cycles of visible light irradiation. For both electrodes, the photocurrent density increased rapidly when irradiated with visible light and then dropped sharply when the light was turned off. Importantly, the photocurrent density of the flower-like CeO_2_ NPs/CuO NPs catalyst (40.75 μA cm^−2^) was about 10 times higher than that of the CuO NPs catalyst (4.28 μA cm^−2^) under visible light irradiation, indicating that charge separation much more effective on the CeO_2_ NPs/CuO NPs. This data provides further evidence that CeO_2_/CuO heterojunction construction was effective in enhancing the light absorbing and availability of electrons and holes for photoreactions (by effectively facilitating charge separation). Further, the photocurrent density of CeO_2_ NPs/CuO NPs electrode appeared to be quite stable over the three on–off cycles, suggesting that the electrode had stable performance.

The XPS valence band spectrum for the flower-like CeO_2_ NPs/CuO NPs catalyst is shown in Fig. 4b. The spectrum could be fitted using two peaks at 3.97 and 5.39 eV, corresponding to valence oxygen states CeO_2_ and CuO. The position of the VBM was obtained directly from the spectrum using a linear extrapolation, yielding a VBM of 1.47 eV. Since the band gap of the flower-like CeO_2_ NPs/CuO NPs catalyst was estimated to be around 2.10 eV (Fig. 2c), the CB position of CeO_2_ NPs/CuO NPs catalyst was thus around − 0.63 eV.

### Photoelectrocatalytic Reduction of CO_2_

**Fig. 5:**
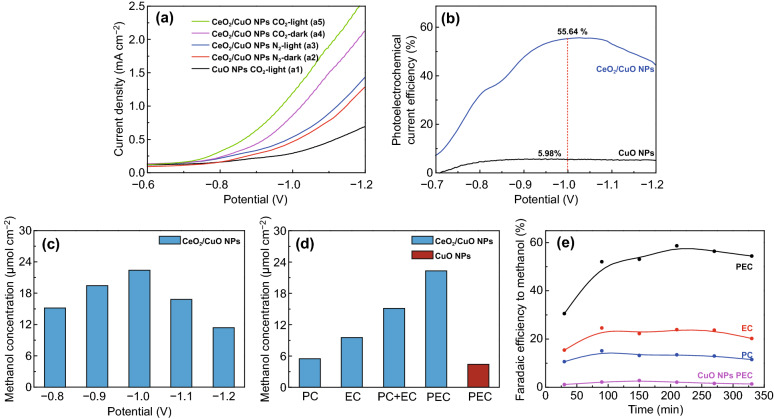
**a** Linear sweep voltammetry curves for different electrodes under light illumination and in the dark. The data were collected in a 0.1 mol L^−1^ KHCO_3_ solution, under N_2_ or CO_2_ purging. **b** Photoelectric conversion efficiencies for different electrodes. **c** The methanol concentration at different potentials following 6.5 h of light illumination for the CeO_2_ NPs/CuO NPs catalyst. **d** The methanol concentration after 6.5 h for PEC, EC, and PC reduction of CO_2_ at − 1.0 V. **e** Faradaic efficiency for CO_2_ reduction to methanol under PEC, EC, and PC conditions at − 1.0 V

Having characterized the CuO/NPs and flower-like CeO_2_ NPs/CuO NPs catalyst in detail, we then examined the performance of these two electrodes for PEC reduction CO_2_. Figure 5a shows linear sweep voltammetry (LSV) curves for each electrode in a 0.1 mol L^−1^ KHCO_3_ solution, under N_2_ or CO_2_ purging. In each case, the increase in the current observed as the potential is made more negative is due to the reduction of water and/or the catalytic reduction of CO_2_. The current response of the CeO_2_ NPs/CuO NPs catalyst increased under light irradiation, under both a N_2_ purge (cf. curves in Fig. 5a_2_, a_3_) and a CO_2_ purge (cf. curves in Fig. 5a_4_, a_5_). Results indicate that the CeO_2_ NPs/CuO NPs catalyst had good performance for PEC CO_2_ reduction. Further, the PEC CO_2_ reduction performance of the CeO_2_ NPs/CuO NPs catalyst was much better than that of the CuO NPs catalyst under light irradiation and with a CO_2_ purge (cf. curves in Fig. 5a_5_, a_1_). And then the net current density $(i_{{CO_{2} }} - i_{{N_{2} }} )$ of the CeO_2_ NPs/CuO NPs with light and without light was compared (Fig. S4), the current density of light is much higher than dark one. It is more validated that the CeO_2_ NPs/CuO NPs have more excellent ability for PEC reduction CO_2_. The photoelectric conversion efficiencies of the flower-like CeO_2_ NPs/CuO NPs and CuO NPs catalysts are shown in Fig. 5b. The CO_2_ to CH_3_OH conversion efficiency on the CuO NPs/electrode at the applied potential of − 1.0 V (vs SCE) was only 5.98%, whereas the efficiency for the same conversion on the CeO_2_ NPs/CuO NPs catalyst was 55.64% (i.e., around nine times higher).

The products of PEC CO_2_ reduction on the CeO_2_ NPs/CuO NPs catalyst were analyzed by gas chromatography. At externally applied bias voltages ranging from − 0.8 to − 1.2 V, the only carbon-containing reduction product detected was methanol. Figure 5c shows the methanol yield over the CeO_2_ NPs/CuO NPs catalyst at different applied potentials. As the applied voltage was increased up to − 1.0 V, the yield of methanol increased, reaching 22.32 μmol cm^−2^ at − 1.0 V over 6.5 h (corresponding to a methanol production rate of 3.43 μmol cm^−2^ h^−1^). As the potentials continued to increase, at higher potentials, the yield of methanol decreased. Results suggest that in the potential range from − 0.8 to − 1.0 V, the catalytic reduction of CO_2_ is the dominant reaction occurring on the electrode. At more negative potentials, the hydrogen evolution reaction starts to compete with electrocatalytic CO_2_ reduction.

Figure 5d shows comparison of the methanol yields for CO_2_ reduction to methanol on the flower-like CeO_2_ NPs/CuO NPs catalyst under PEC, EC, and PC regimes after 6.5 h of testing. Methanol yields decreased in the order PEC (22.32 μmol cm^−2^) > EC (9.51 μmol cm^−2^) > PC (5.53 μmol cm^−2^). Thus, the methanol yield by PEC reduction of CO_2_ was 2.35 and 4.04 times higher than the yields obtained by EC and PC. Further, the methanol yield by PEC on the CeO_2_ NPs/CuO NPs catalyst (22.32 μmol cm^−2^) was around 5 times higher than the methanol yield by PEC on the CuO NPs electrode (4.37 μmol cm^−2^) for a reaction time of 6.5 h at a potential of − 1.0 V, indicated that CeO_2_ addition greatly improved the PEC performance of the CeO_2_ NPs/CuO NPs catalyst. The Faradaic efficiency for CO_2_ reduction to methanol on the flower-like CeO_2_ NPs/CuO NPs catalyst under different conditions (i.e., PEC, EC, and PC) in 0.1 mol L^−1^ KHCO_3_ solution under a CO_2_ purge is shown in Fig. 5e. The Faradaic efficiency for PEC was 58.67%, significantly higher than that realized by EC (24.57%) and PC (15.08%). Again, this reflects the action of both EC and PC processes under the PEC regime, which act cooperatively to increase the availability of electrons for CO_2_ reduction. As expected, the PEC Faradaic efficiency of the CeO_2_ NPs/CuO NPs catalyst for CO_2_ reduction to CH_3_OH at − 1.0 V was superior to that of the CuO NPs catalyst. Furthermore, the Faradaic efficiency of the CeO_2_ NPs/CuO NPs catalyst remained ~ 55% at − 1.0 V over 6.5 h, implying that the heterojunction electrode had exceptional stability.

Considering all of the above, it can be concluded that the CeO_2_ NPs/CuO NPs catalyst developed in this work demonstrated remarkable performance and stability for PEC CO_2_ reduction to methanol. The electrode benefitted from the following synergies: (1) The direct electrodeposition of flower-like CeO_2_ nanoparticles on the CuO NPs/Cu foil catalyst ensured intimate contact between the CeO_2_ and CuO components, thus forming intimate p–n heterojunctions for electron transfer; (2) The flower-like CeO_2_ offered an abundance of active sites for CO_2_ adsorption and reduction, while also minimizing electron–hole pair recombination in CeO_2_ under light irradiation due to the short electron transfer distance to the CeO_2_ surface distance; (3) The combination of p-type CuO and n-type CeO_2_ increased the carrier concentration in the CeO_2_ NPs/CuO NPs catalyst by 10^8^ times relative to the CuO NPs catalyst, which greatly enhanced CO_2_ reduction performance; and (4) The CeO_2_ NPs/CuO NPs catalyst possessed good optical absorption characteristics under visible light, with the photoexcitation of both CeO_2_ and CuO, as well as electron transfer from CuO to CeO_2_ (matched by hole migration from CeO_2_ to CuO), generating an abundance of electrons at the CeO_2_ surface for CO_2_ reduction. Thus, when light and external voltage (− 1.0 V) were simultaneously applied to the CeO_2_ NPs/CuO NPs catalyst in a 0.1 M KHCO_3_ solution purged with CO_2_, the reduction of CO_2_ to CH_3_OH occurred very efficiently evidenced by a Faradaic efficiency of almost 60% at − 1.0 V (using protons generated by water oxidation at the anode). Results encourage the development of p–n junction PEC systems for CO_2_ reduction to oxygenate and fuels.

## Conclusions

Electrodeposition of flower-like CeO_2_ NPs significantly enhanced the performance of a CuO NPs/Cu catalyst for PEC CO_2_ reduction to methanol under visible light irradiation. The system benefitted from the creation of intimate p–n heterojunctions between p-type CuO and n-type CeO_2_. Under both an applied electric potential of − 1.0 V, or visible light irradiation, electrons migrated from CuO nanoparticles to the flower-like CeO_2_ nanoparticles, resulting in an abundance of electrons on the surface of the CeO_2_ nanoparticles for CO_2_ reduction to methanol. Under visible light illumination, the yield of methanol for the CeO_2_ NPs/CuO NPs/Cu catalyst at − 1.0 V in a CO_2_–saturated 0.1 M KHCO_3_ solution was 3.44 μmol cm^−2^ h^−1^ (cf. the CuO NPs/Cu catalyst, for which the methanol production rate was only 0.67 μmol cm^−2^ h^−1^). Further, under these conditions the CeO_2_ NPs/CuO NPs/Cu catalyst displayed a high Faradaic efficiency for CO_2_ reduction to CH_3_OH (~ 60%) and excellent stability. These findings are significant for the future development of catalytic systems for CO_2_ reduction, demonstrating that a simple catalyst modification (i.e., CeO_2_ electrodeposition) can dramatically enhance carrier concentrations and the availability of electrons for PEC reduction of CO_2_ to valuable commodity chemicals such as methanol.

## Electronic Supplementary Material

Below is the link to the electronic supplementary material.

Supplementary material 1 (PDF 234 kb)
