# Supplementary material for: Highly Efficient Photoelectrocatalytic Reduction of CO2 to Methanol by a p–n Heterojunction CeO2/CuO/Cu Catalyst
Source: Nanomicro Lett. 2020 Jan 9;12:18. doi: 10.1007/s40820-019-0354-1 (PMC7770658; doi:10.1007/s40820-019-0354-1)
Supplement: Supplementary file 1 — Supplementary material 1 (PDF 234 kb) [file 40820_2019_354_MOESM1_ESM.pdf]

Nano-Micro Letters

Supporting Information for

Highly Efficient Photoelectrocatalytic Reduction of CO_2 to Methanol by a p-n Heterojunction CeO_2/CuO/Cu Catalyst

Zhengbin Pan[1, 2, #], Ershuan Han[1, 2, #], Jingui Zheng[2], Jing Lu[2], Xiaolin Wang[2], Yanbin Yin[2], Geoffrey I.N. Waterhouse[2, 3, *], Xiuguo Wang[1, *], Peiqiang Li[2, *]

[1]Tobacco Research Institute of Chinese Academy of Agricultural Sciences (CAAS), Qingdao 266101, People’s Republic of China

[2]College of Chemistry and Material Science, Shandong Agricultural University, Tai’an 271018, People’s Republic of China

[3]School of Chemical Sciences, The University of Auckland, Auckland 1142, New Zealand

[#]Zhengbin Pan and Ershuan Han contributed equally to this work

*Correspondence authors. Email: wangxiuguo@caas.cn (Xiuguo Wang); chem_carbon@outlook.com (Peiqiang Li)

Supplementary Figures

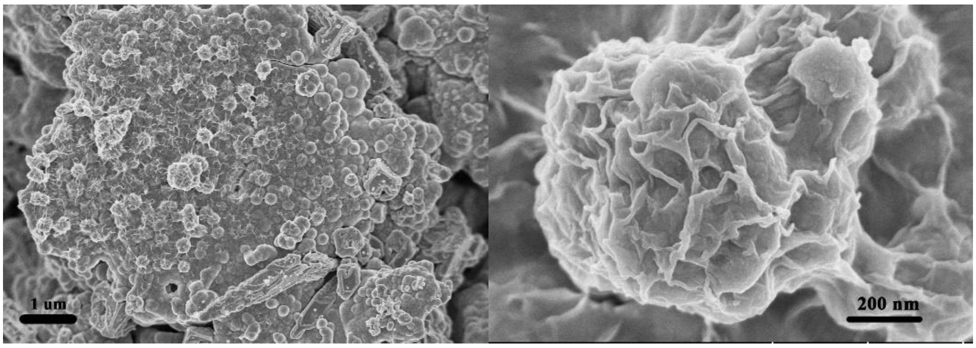

Fig. S1 SEM images of flower-like CeO_2 NPs/CuO NPs

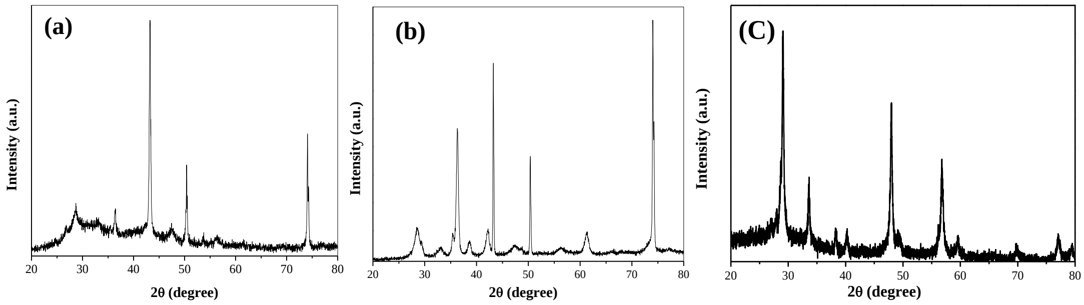

Fig. S2 XRD patterns for **a** CuO **b** flower-like CeO_2 NPs/CuO, and **c** CeO_2 NPs

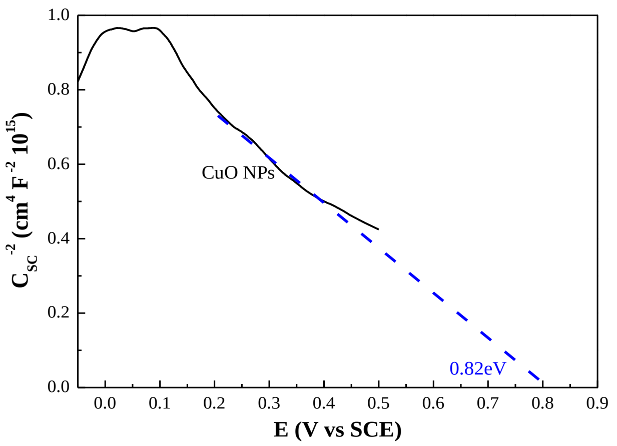

Fig. S3 Mott-Schottky plots for CuO NPs

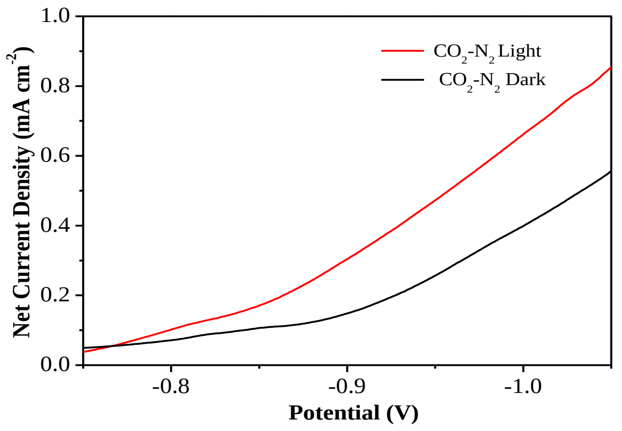

Fig. S4 The net current density of CeO_2 NPs/CuO NPs

Table 1 The XPS results of Ce 3d

Peak Label	Ce Valence state	Peak Characteristics	Binding energy (eV)	Ratio (%)
μ‴	Ce^{4+}	$3d_{3/2}$	912.50	9.84
μ″	Ce^{4+}	$3d_{3/2}$	904.09	5.87
μ′	Ce^{3+}	$3d_{3/2}$	900.46	17.64
μ	Ce^{4+}	$3d_{3/2}$	897.17	17.31
v‴	Ce^{4+}	$3d_{5/2}$	894.29	9.58
v″	Ce^{4+}	$3d_{5/2}$	885.14	5.72
v′	Ce^{3+}	$3d_{5/2}$	881.75	17.18
v	Ce^{4+}	$3d_{5/2}$	878.46	16.86
Ce^{3+}/ (Ce^{4+} + Ce^{3+})				34.82
